# Evaluation of a depression care model for the hill tribes: a family and community-based participatory research

**DOI:** 10.1186/s12888-023-05058-3

**Published:** 2023-08-04

**Authors:** Onnalin Singkhorn, Pawadee Hamtanon, Katemanee Moonpanane, Khanittha Pitchalard, Rachanee Sunsern, Yosapon Leaungsomnapa, Chananan Phokhwang

**Affiliations:** 1 School of Nursing, Mae Fah Luang University, Chiang Rai Province, Mueang Chiang Rai, Thailand; 2 Center of Excellence for the Hill Tribe Health Research and Training, Mae Fah Luang University, Mueang Chiang Rai, Thailand; 3 Nursing Faculty, Thaksin University, Phatthalung, Thailand; 4 School of Health Science, Mae Fah Luang University, Mueang Chiang Rai, Thailand; 5 Ministry of Public Health, Phrapokklao Nursing College, Faculty of Nursing, Praboromarajchanok Institute, Mueang Chanthaburi, Thailand; 6 Nursing Faculty, Rajabhat University, Surat Thani, Thailand

**Keywords:** Community-based participatory research, Depression, Ethnic group, Hill tribes, Minority group

## Abstract

**Background:**

Even though, there is a particularly high prevalence of depression among individuals from the hill tribes in northern Thailand, they are unable to receive appropriate intervention due to cultural, transportation, communication, and legal barriers. Using community-based participatory research (CBPR), a depression care model was developed for the hill tribe population. The effectiveness of this model was examined using questionnaires, observations, focus groups, and in-depth interviews.

**Methods:**

Participants include people with depression (*n* = 17) who were chosen based on their mild to moderately severe depression scores on the Patient Health Questionnaire 9-item (PHQ-9 scores of 5–19) and their caregivers (*n* = 5). The in-depth interview was conducted to distinguish the selected participants into two groups. The first group, the self-help group program, consisted of 12 participants endorsing negative thoughts about themselves and inappropriate problems solving. The second group, the family camp program, had ten participants, including five patients with family-related issues and their family members. Subjects separately participated in either the self-help or the family groups over three weeks. They completed the PHQ-9 at the beginning and end of the intervention. Questionnaires, observations, focus groups, and in-depth interviews were used to evaluate the effectiveness of the model. Content analysis was used to examine the qualitative data. Wilcoxon signed-rank test was used to analyze the changes in the severity of depression before and after participation in the intervention.

**Results:**

The depression scores on the PHQ-9 of 12 participants improved significantly (11.92 ± 1.08 *vs.* 3.08 ± 0.51; *p* = 0.002) following participation in the self-help group. Increased self-esteem and improved interpersonal relationships were reported by participants in the self-help group program during interviews. There was no significant difference in the depression scores of 10 participating in the family camp program (6.00 ± 3.83 to 5.30 ± 3.56; *p* = 0.161).

**Conclusion:**

A model for depression care was tested in a hill tribe community, and its effectiveness was clearly observed. The developed model can be applied to other hill tribe communities in northern Thailand to improve depression care.

## Introduction

Multimorbidity and depression have been highlighted as one of significant challenges to healthcare systems, especially in low-and middle-income countries. A meta-analysis across 43 low-and-middle-income countries estimated that the pooled odd ratio (OR) for multimorbidity and depression was 3.3 (95% CI 2.98–3.57) [1]. Depression is a risk factor for mild cognitive impairment (MCI), which affected competency and decreased quality of life, especially in the subdomains of happiness, life freedom, decision-making, interpersonal relationship, and life satisfaction [2]. Thailand ranks fourth in Southeast Asia in terms of the number of recorded incidents of depression. Even though more than 14 million persons at risk in Thailand have been assessed for depression and have received mental health education, about half of individuals with depressive disorders had access to standard care and approximately 1.7 million patients had received psychosocial interventions [3]. Despite the fact that the rates of emotional illness in Thailand continue to climb, a recent study has reported that there are 882 psychiatrists and psychiatric residents in the country [4]. It is insufficient to help the entire population, especially in rural areas.

According to the 2019 report from the WHO, there were approximately three million hill tribe members in Thailand [5]. Around one-third of these populations live in the rural border areas of Thailand, including Chiangrai, Chiangmai, Mae Hong Son, Lamphun, Nan, and Tag, as well as Myanmar and Laos [6]. The prevalence of depression varies among different ethnicities and countries [7, 8]. Rates range from around 10% in the Black Irish population to as high as 50% among Iranians [7, 8]. In our earlier research, we found a significantly high prevalence of depression among hill tribe individuals in Chiang Rai province. Among the hill tribe adults, the prevalence of depression is approximately 39% [9], whereas among other populations in Thailand, it ranged from 6 to 20% [10, 11]. Additionally, several factors have hindered the hill tribes from accessing appropriate psychosocial health services. These factors include cultural practices and beliefs, inadequate income, low education, language barriers, lack of access to roads [9], absence of Thai identification documents [12], social inferiority, and stigma associated with illegal methamphetamine and opium use, among others [13]. Following the studies of other populations, where the family unit plays a crucial role in supporting patients with mental illness [14–16], we found that family-related factors, such as being female, 50 years or older, married, Christian, and living with a relative, were associated with depression in hill tribe individuals [17]. However, prior research has not established a unique method focusing on family and community involvement to support people with mental illnesses in Thailand, particularly those from ethnic backgrounds, including hill tribes. To address this healthcare issue, there is an urgent need for effective, low-cost community-based psychosocial prevention and interventions tailored to the specific needs of the hill tribe communities.

To strengthen a community's ability to address major health challenges, particularly depression, the community-based participatory research (CBPR), an academic-community partnership study [18], was employed to develop a depression care model called ‘the SMILE model’ for hill tribe members with depression. The SMILE is an acronym that represents the following key components: (S)stakeholders' readiness to care for people from hill tribes with depression, (M)motivation of people from hill tribes with depression to change their behaviours, and that of their family and community in depression care, (I)interpersonal relationships within the hill tribes, (L) life and community assets of the hill tribes, and (E)empowerment of the community members. This model has been developed based on WHO's Innovative Care for Chronic Condition (ICCC) framework [19]. Collaboration between mental healthcare practitioners, public health providers, academic institutions, local administration organizations, families, and the community is required to create an effective collaborative care model for depression and offer support for those who are depressed. Previous studies [20–22] have demonstrated that community-based preventive programs effectively reduced certain mental health symptoms, including depression, while also increasing self-esteem and social support. Therefore, in this current research, our aim is to further examine the efficacy of our collaborative depression care intervention, named the 'SMILE model,' which was specifically developed for hill tribe members with depression [23]. This unique model was applied to volunteers with mild to moderate depression from Akha, Mien, and Lahu communities, and its effectiveness was assessed by analysing the changes in their PHQ-9 scores.

## Methods

### Study design

As described earlier [23], we acquired qualitative data, created, and evaluated the SMILE model in the same Ban Lao-fu Village community using information from the quantitative survey, the ICCC framework, and family-community engagement. The Look, Think, and Act methods were employed as spiral steps in three phases of the model development, which followed the Stringer idea [24]. The family-community-participation project was conducted to develop the SMILE model by surveying the depression care system, current problems, and needs of patients with depression, their relatives, healthcare providers, and the community from the Ban Lao-fu Village of the Pa-Tung sub-district, Maejan District, Chiang Rai Province, where more than 300,000 hill tribe members lived [9].

To establish the trustworthiness of this present work, methodological triangulation was employed, utilizing both qualitative and quantitative methods, along with conducting both focus groups and individual interviews to collect data. Furthermore, data triangulation was also used to increase the credibility of this research. The information obtained from the participants and stakeholders was cross verified by comparing the results obtained from multiple sources, including their family members, village chiefs, village health volunteers, etc. The depression care model development and testing were conducted in Ban Lao-fu Village in January 2019. To evaluate the effectiveness of the SMILE model, the self-help group and the family camp programs ran concurrently for three weeks in September 2019.

### Setting and samples

This study was conducted in Ban Lao-fu village. This village consists of 2,402 people from 575 families from four hilltribe groups include Akha, Mien, Lisu, and Lahu. Participants (*n* = 12) who expressed automatic negative thoughts during clinical interviews were assigned to the self-help group program based on their mild to moderately severe depression scores on the Patient Health Questionnaire 9-item (PHQ-9 scores of 5–19). Five participants (*n* = 5) and their relatives (*n* = 5) were allocated to the family camp program after clinical interviews revealed a family conflict and mild to moderately severe depression scores. The purposive sampling technique was employed in this study. Inclusion criteria for the volunteers were age ≥ 40 years, had PHQ-9 scores of 5–19, and were able to read and understand the Thai language. The subjects were excluded from our study if they had been diagnosed with psychotic disorders or currently had depression with psychotic symptoms or were on antidepressants or medication affecting sleep.

### Ethical considerations

The Human Research Ethics Committee at the Chiangrai Provincial Public Health Organization (CRPPHO 6/2562, January 7, 2019) approved this study. All participants were given detailed information about the study and verbally or in writing agreed to participate. They also verbally authorized the audio recordings of the interviews, focus groups, and program participation. Informed consent from legally authorized representatives/guardians for illiterate participants has been obtained before the participation. Their rights as study subjects were protected in accordance with the principles of the Helsinki Declaration.

### Intervention allocation and outcome measurement

Participants suffering from depression were assigned to either the self-help group or the family camp programs. Depression stigma and cultural beliefs that negatively impacted depression care was also discussed with these participants. Changes in the depression scores based on the PHQ-9 were used as a primary outcome of this study. Stakeholders' levels of satisfaction with the SMILE model's implementation were measured through open-ended questions asked in feedback interviews.

Twelve participants with depression and showed automatic negative thoughts (Table 1) were assigned to the self-help group program based on the SMILE model’s concept and reality therapy [25] using the WDEP (wants, doing, evaluation, and planning) technique [26]. This self-help group program emphasized motivation, interpersonal relationships, life and community assets and empowered patients to care for themselves and seek help and care from their families, community, and health service centre. The program lasted three weeks, with two weekly activity sessions lasting 60–90 min each.

**Table 1:** Participant characteristics

Characteristics of participants	N or Mean	% or SD
**Participants in Self-help group program (*n* = 12)**		
Gender (male/female)	3/9	25/75
Age (in years)	51.92 (min = 44/max = 58)	4.68
Marital status		
Single	1	8.33
Married	9	75.00
Divorced	2	16.67
Education		
Illiterate	4	33.33
Primary school	8	66.67
Sufficient income (yes/no)	3/9	25.00/75.00
**Participants in Family camp program (*n* = 10)**		
Gender (male/female)	5/5	50/50
Age (in years)	50.30 (min = 40/max = 59)	5.33
Marital status		
Married	10	100.00
Education		
Illiterate	4	40.00
Primary school	6	60.00
Sufficient income (yes/no)	2/8	20.00/80.00

For individuals with family issues, five participants and their family members (*n* = 5) attended the family-group therapy according to the SMILE model’s concept and Satir's model [27]. The Satir model for family therapy focused on intrapsychic (internal experience), interactive (conflict resolution), and family-of-origin-system (family reconstruction for resource and transformational change) [28]. In an intervention study on Thai patients with schizophrenia and their families, the Satir model family therapy compared to group psychoeducation helped decrease the severity of symptoms, improve the patient’s social functioning, and increase the family members' self-esteem [29]. Our Satir family group therapy program focused on motivation, interpersonal relationships, and empowered families to care for patients with depression. The program lasted three weeks, including two weekly 60–90-min activity sessions.

### Research instruments

The Patient Health Questionnaire 9-item: PHQ-9 [30], which is a standard instrument, was used to measure the depression level severity of participants as a screening tool and for program evaluation. The information for the index of validity, reliability, sensitivity and specific of this instrument were found to be 0.56 (*P* < 0.001; compared with the HAM-D), 0.79 (Cronbach's alpha), 0.53, and 0.98, respectively [30]. In addition, our previous work revealed the index of reliability of 0.74 for the PHQ-9 [9]. A semi-structured interview guide with three open-ended questions was used to explore the experiences of participants and families with the self-help and family groups.

### Data analysis

The Wilcoxon signed-rank test was used to compare the depression scores of patients before and after program interventions due to the small sample size and non-normal distribution of the data. Content analysis was used to analyse qualitative data.

## Results

### Self-help group program

Of the 12 participants, 75% were female. The participants’ ages ranged from 44 to 58 years old, with a mean age of 51.92 (SD = 4.68). Seventy-five percent of the participants were married. Most participants had a primary school education (66%), and 75% had insufficient income (Table 1).

We found that depressive symptoms improved among individuals participating in the self-help group program (Table 2). In interviews, participants in the self-help group felt better, reported higher self-esteem, and more confidence in taking care of themselves. Participants also reported seeking help and care from their families, the community, and the healthcare centre. In interviews, they also reported more social interaction with the community and healthcare providers. We used a Wilcoxon signed-rank test to assess the depression score as a pre-and-post-test of the self-help group. The depressive score significantly decreased after participation in the self-help group (Pre: 11.91 ± 1.08, Post:3.08 ± 0.51; *p* = 0.002).

**Table 2:** Change in depression scores on the PHQ-9 before and after participation in the self-help group and the family camp programs

**Module**	**Median score**		**Median change**	**Signed rank test**	***p*-value**
	**before training**	**after training**	**in percentage points (Δ)**	**statistic (Z)**	
**Participants in the self-help group program (*n*=12)**					
Depression	12.00	3.00	-9.00	3.086	0.002
**Participants in the family camp program (*n*=10)**					
Depression					
- *Total* (*n*=10)	4.40	3.00	-1.40	-1.403	0.161
- *Patients* (*n*=5)	10.00	8.00	-2.00	-0.962	0.336
- *Family members (n=5)*	3.00	2.00	-1.00	-1.342	0.180

### Family camp program

Of the 10 participants, including five patients and their family members, the ages ranged from 40 to 59 years old, with a mean age of 50.30 years (SD = 5.33). All of the participants were married. Most participants had a primary school education (60%), and 80% had insufficient income (Table 1). The depression scores on PHQ-9 of the family members (*n* = 5) ranged from 2 to 3. We found that the family camp program activities were not suitable due to the nature and cultures of the hill tribes (Table 2). Based on interviews, most people from the hill tribes with depression and their families reported feeling uncomfortable sharing their thoughts and feelings with others due to cultural beliefs. They were also not concerned about the importance of discussing depression with their family members. For depression and issues in the family, they believed that only men, who were the head, make decisions. This contributed to the symptoms of depression experienced by women. The husbands of some women participating in the program were open to discussing the role of the power differential in maintaining symptoms of depression in their wives but were unwilling to change the power hierarchy. Furthermore, participants felt that family members should not say anything regarding the family to others outside the family. Moreover, only ten family members from five families participated in the program, as most families were busy. Families were impoverished and had to work as laborers from Monday to Saturday for their daily income. They usually were free on Sundays, and most attended church. There was no significant difference in the depression scores of people participating in the family camp program (6.00 ± 3.82 to 5.30 ± 3.56; *p* = 0.161).

### The content analysis of qualitative data

Using content analysis, the qualitative data that reflects the efficacy of the SMILE model was analyzed and categorized into three main categories, including the satisfaction of the participants with the SMILE model, the improvement of participants' self-esteem and confidence, and the interpersonal relationships among the participants, their relatives, and the village help volunteers.

According to the interviews, the participants expressed satisfaction with the implementation of the SMILE models. It was found that participants experienced stigma related to having depression. However, the introduction of the SMILE model helped reduce their stigma by improving the knowledge associated with depression among participants, community leaders, their relatives, and the village help volunteers. The participants and their caregivers also mentioned that the cost and time saving offered by the SMILE model were advantages of this collaborative care program.

“I feel that both my family and I have a better understanding of my symptoms, and I have hope that they can be cured.” (Participant 1)

“I understand that the person with depression is ill and not possessed by evil spirits.” (Participant 2)

“I am happy that the model improves the system for taking care of individuals with depression. Furthermore, we no longer need to go to the hospital because we can provide care for those with depression through the system established in our community.” (Participant 3)

Furthermore, the participants' self-esteem and confidence improved after they completed the self-help group program indicated above.

“I will never again let the words of others poison my heart.” (Participant 4)

“For once, I feel like my happiness is in my hands.” (Participant 5)

The introduction of the SMILE model also enhances participant’s interpersonal relationships both in the self-help group program and the family camp program.

“I am excited to participate in the group and share our ideas with the others.” (Participant 6)

“We have created the Line group to facilitate communication and the exchange of information among participants.” (Participant 7)

## Discussion

In the present study, the self-help group program, which is one component of the SMILE model, effectively improved the depression scores of hill tribe patients. However, no significant improvement was detected for participants in the family camp program.

The SMILE model has been successfully developed by incorporating the CBPR approach with the ICCC framework specifically for the hill tribe population [23]. Based on intensive review articles [31, 32], CBPR studies offer new perspectives and are inclined to approach mental health promotion differently than traditional methods that focus on illness. Furthermore, there is a growing demand for more culturally relevant and comprehensive research approaches and methods from community leaders, nurses, public health technical officers, health volunteers, and potential research participants, especially when working with minorities and underserved populations [31, 32]. Previous articles indicate that collaborative care interventions for psychiatric disorders have consistently shown success in improving key outcomes in both research and clinical intervention studies, enhancing the quality of patient care, and improving population health [33–36]. Furthermore, cost analyses also suggest that this model is cost-effective [37]. For the first time in this study, the effectiveness of implementation processes and adaptation of this collaborative care model to align with the clinical realities of general practice for individuals from the hill tribe with depression has been proven. Therefore, it is important to note the SMILE model, precisely the self-help group program developed by our team [20], as a unique, cost-saving, and effective program for hill tribe individuals with depression.

The depressive symptoms of people from hill tribes participating in the self-help group were improved. This improvement is likely due to the fact that the main activity of the self-help group program was counselling. Counselling involves helping those with depression change their way of thinking, feeling, and behaving. Furthermore, counselling was a goal-based collaboration process between interventionists and those with depression to set goals, develop strategies, and plan to achieve the goals. At the start of the program, the interventionist built a good relationship between the group leader and members, and within group members to create a culture of trust. All group members created their rules to participate in counselling with acceptance and empathy. During counselling, the group leader used active listening, silence, open-ended questions, and exploration to facilitate [38]. All these techniques helped those with depression to trust, share their feelings, thoughts, and behaviours, and deal with their problems [39]. Furthermore, praise when members offer their thoughts in the group. These compliments made members feel pleased and like they were a part of the group [40], and they were the cause of less depression [41].

Most people from hill tribes with depression were uncomfortable sharing their thoughts and feelings with family members due to cultural beliefs. Thus, the development of a family relationship based on the Satir model, a main component of the family camp program, might be unsuitable for them. A possible reason might be related to the program concept, which focused on expressing feelings for each other and encouraged family members to share their thoughts and feelings, especially on the topic of family relationships, which was too sensitive [42]. In addition, the Satir Model was limited in dealing with family issues, particularly sexual abuse and family violence. Within the hill tribes, the head of the family-controlled family members. This hierarchy is common in Southeast and South Asia, which are characterized by a high level of gender inequality. A preference for sons and an aversion to daughters are common in many countries. The man is the heir of the family, but the woman, once married, is the property of her husband [43]. Families in the hill tribe communities had hierarchical and collectivist values. Therefore, a discussion of the family roles and expectations was unsuitable. Moreover, most activities during the family camp program were specific in teaching communication skills (for example, wording, posture, and body movement). Unfortunately, these activities might not be suitable for traditional hill tribe families, who strongly believe in their culture of keeping secrets (not sharing anything with others). It will be a challenge for health providers and researchers to create a new communicative strategy for hill tribe family members. Further culturally sensitive and focused research is required.

The SMILE model can be effectively applied to address depression in the hill tribe communities. However, model users need to consider cultural sensitivity and language barriers and adjust the model to the hill tribes' contexts. Motivation to learn the language and to have hope for the future is also the key to success in depression care at the patient-family micro level. It is also critical to strengthen the community's roles in providing care for patients with depression through VHVs. Aside from adhering to the Ministry of Public Health's policies and guidelines, stakeholders, particularly health care providers, and authorities, should provide mental health care to hill tribes suffering from depression in accordance with policies and guidelines established explicitly by the community and implemented in the community. Further research should explore cultural beliefs and practices of self-care of patients, families, and communities as it relates to caring for depression among other hill tribes. In addition, future studies should examine the effectiveness of the "SMILE model" in other hill tribe groups with depressions.

One of the main limitations of this study is the small sample size. Only twelve participants completed the intervention. For the family camp, only five families participated in the camp due to their economic backgrounds and work. They did not think the camp was important because they needed to work for their family's living. Therefore, to gain better family and community participation in a project or activity, it should be conducted on Sunday afternoons, when most are free, and the church services finish. In addition, individual family therapy instead of group therapy, should be provided to the patients and families due to depression and family issues being very culturally and familial sensitive.

## Conclusion

The ‘SMILE model’ is a depression care model based on family and community participation and designed for people from hill tribes with depression. It is a well-formulated, framework-based, and effective model for addressing depression in hill tribe communities. The model implementation led to positive outcomes for people from hill tribes with depression, their families, and the community.

## Data Availability

Due to the ethical concern with participant’s data and privacy, the datasets obtained and/or analyzed during the current study are not publicly available. The information and materials discussed in the manuscript are, nevertheless, accessible upon the relevant author's justifiable request to the corresponding author (Onnalin Singkhorn; onnalin.sin@mfu.ac.th).

## Abbreviations

CBPR: Community-based participatory research
VHV: Village Health Volunteer
OR: Odd ratio
MCI: Mild cognitive impairment
WHO: World Health Organization
ICCC: Innovation Care for Chronic Condition framework
CRPPHO: Chiangrai Provincial Public Health Organization
PHQ-9: Patient Health Questionnaire 9-item
HAM-D: Hamilton Depression Rating Scale
WDEP: Wants, doing, evaluation, and planning technique of reality therapy
